# A Pilot Study on the Potential Pathological Link Between Oxidative Stress Markers and Renal Function in People Living with HIV

**DOI:** 10.3390/ijms262311429

**Published:** 2025-11-26

**Authors:** Joel Choshi, Haskly Mokoena, Helen C. Steel, Brian T. Flepisi, Kabelo Mokgalaboni, Wendy N. Phoswa, Gerald Maarman, Sihle E. Mabhida, Bongani B. Nkambule, Phiwayinkosi V. Dludla, Sidney Hanser

**Affiliations:** 1 Department of Physiology and Environmental Health, University of Limpopo, Sovenga 0727, South Africa; haskly.mokoena@ul.ac.za; 2 Department of Immunology, University of Pretoria, Pretoria 0001, South Africa; helen.steel@up.ac.za; 3 Department of Pharmacology, University of the Witwatersrand, Braamfontein, Johannesburg 2050, South Africa; brian.flepisi@wits.ac.za; 4 Department of Life and Consumer Sciences, College of Agriculture and Environmental Sciences, University of South Africa, Florida Campus, Roodepoort 1710, South Africa; mokgak@unisa.ac.za (K.M.); phoswwn@unisa.ac.za (W.N.P.); 5 CARMA: Centre for Cardio-Metabolic Research in Africa, Division of Medical Physiology, Department of Biomedical Sciences, Faculty of Medicine & Health Sciences, Stellenbosch University, Cape Town 8000, South Africa; gmaarman@sun.ac.za; 6 Non-Communicable Diseases Research Unit, South African Medical Research Council, Tygerberg 7505, South Africa; sihle.mabhida@mrc.ac.za; 7 School of Laboratory Medicine and Medical Sciences, University of KwaZulu-Natal, Durban 4000, South Africa; nkambuleb@ukzn.ac.za (B.B.N.); dludlap@unizulu.ac.za (P.V.D.); 8 Department of Biochemistry and Microbiology, University of Zululand, KwaDlangezwa 3880, South Africa; 9 Department of Physiology, School of Medicine, Sefako Makgatho Health Science University, Ga-Rankuwa 0208, South Africa

**Keywords:** antiretroviral therapy, oxidative stress, people living with HIV, renal function

## Abstract

Human immunodeficiency virus (HIV) infection remains a major health burden in Sub-Saharan Africa, despite the widespread use of antiretroviral therapy (ART). Oxidative stress contributes to HIV-related comorbidities, including renal dysfunction. However, the role of oxidative stress in kidney impairment among people living with HIV (PLWH) is not fully understood. This cross-sectional study included PLWH on ART (*n* = 80), PLWH without ART (*n* = 27), and people not living with HIV (PNLWH) (*n* = 44). Oxidative stress was measured by serum malondialdehyde (MDA), superoxide dismutase (SOD) and total antioxidant capacity (TAC), while renal function was assessed using cystatin C-based estimated glomerular filtration rate (eGFR_cystC_). Participants on ART were older (median 43 years) and had higher CD4+ T-cell counts compared to those not on ART. PLWH on ART showed significantly elevated MDA levels compared to PLWH without ART (*p* < 0.001) and PNLWH (*p* = 0.001). There was no difference in superoxide dismutase (SOD) and TAC levels among the groups (*p* = 0.177 and 0.888, respectively). Among PLWH, MDA was higher in those with reduced renal function (eGFR_cystC_ < 90) versus normal function (*p* < 0.05). In PLWH on ART, SOD activity was significantly lower in mild renal impairment (eGFR_cystC_ 60–89) compared to normal function (*p* = 0.017), but no difference was observed in the TAC levels (*p* = 0.883). In PLWH on ART, regression analyses indicated no independent association between MDA and renal function decline, while higher SOD activity independently predicted better renal function (adjusted β = 2.26, *p* = 0.042). Oxidative damage accompanied by the inability of the body’s primary antioxidant defenses may be present at the early onset of renal function decline in PLWH. Superoxide dismutase, as an antioxidant defence enzyme, may be a key contributor to renal health in PLWH on ART. Future studies with larger cohorts and longitudinal designs are needed to clarify these relationships emanating from this pilot study.

## 1. Introduction

Human immunodeficiency virus (HIV) infection remains a major global public health challenge, disproportionately impacting Sub-Saharan Africa (SSA) [1]. Despite the widespread use of effective antiretroviral therapy (ART), an estimated 26 million people in SSA are living with HIV [1]. This includes approximately 8.5 million individuals in South Africa alone, with the prevalence being 14% of the population [2,3]. While ART has dramatically improved viral control and survival, people living with HIV (PLWH) continue to face a growing burden of comorbidities, with renal dysfunction notably adding to the morbidity and mortality of these individuals. This renal impairment is driven, in part, by chronic inflammation and oxidative stress, among other factors [4,5,6].

Oxidative stress, defined as an imbalance between reactive oxygen species (ROS) and the antioxidant defences that neutralize them, is a key pathological mechanism implicated in the progression of many diseases affecting PLWH [7,8]. Its role in renal dysfunction is increasingly recognized, although definitive preclinical and clinical evidence delineating the impact of oxidative damage within HIV-associated kidney injury remains limited [9,10,11]. In HIV-uninfected individuals, oxidative stress is known to accelerate renal decline [12,13]; however, its specific contribution in PLWH remains unclear. This knowledge gap is particularly important for SSA, where the burden of HIV and renal dysfunction is disproportionately high.

Both HIV infection and ART contribute to a pro-oxidant state by disrupting the balance between ROS production and antioxidant capacity [14]. Elevated lipid peroxidation products, such as malondialdehyde (MDA) and 4-hydroxynonenal, combined with reduced levels of antioxidant enzymes, including superoxide dismutase (SOD), catalase, and glutathione, may play a pivotal role in the pathogenesis of renal impairment in PLWH [14,15,16]. However, existing data remain inconclusive, highlighting the need for further investigations into the relationship between oxidative stress markers and renal function deterioration in this population.

The current study addresses this critical gap by exploring the associations between systemic oxidative stress biomarkers and renal function among PLWH on ART. Identifying reliable oxidative stress markers predictive of early renal damage could enhance clinical monitoring and foster the development of novel therapeutic interventions to mitigate kidney disease progression in this vulnerable group. The findings have the potential to inform strategies tailored to the unique challenges faced by PLWH in SSA, ultimately improving long-term health outcomes.

## 2. Results

### 2.1. Demographic and Clinical Characteristics Entailing Baseline Measurements of the Study Population

The baseline characteristics of the study participants, including PLWH on ART (*n* = 80), PLWH not on ART (*n* = 27), and PNLWH (*n* = 44) are summarized in Table 1. Notably, participants on ART had a median age of 43 years, significantly older than those not receiving ART (38 years) and the PNLWH (32 years) (*p* < 0.001). There were no significant differences in gender distribution or use of tobacco across the groups (*p* = 0.583 and *p* = 0.503, respectively). Among PLWH on ART, most individuals had been on treatment for more than three years (*n* = 47, 62.7%). The majority (*n* = 61, 77%) were receiving a regimen consisting of tenofovir disoproxil fumarate (TDF), emtricitabine (FTC) and efavirenz (EFV) (Table 1). The median CD4+ T-cell count was significantly higher in PLWH on ART [441 cells/μL (IQR 248–566)] compared to those not on ART [228 cells/μL (IQR 104–389)] at *p* = 0.004. Notably, the body-mass-index (BMI) of the participants also differed significantly among the groups (*p* = 0.043), with PLWH not receiving ART having the lowest median BMI (Table 1). No differences were observed in systolic or diastolic blood pressure or fasting glucose levels (Table 1).

**Figure 1:**
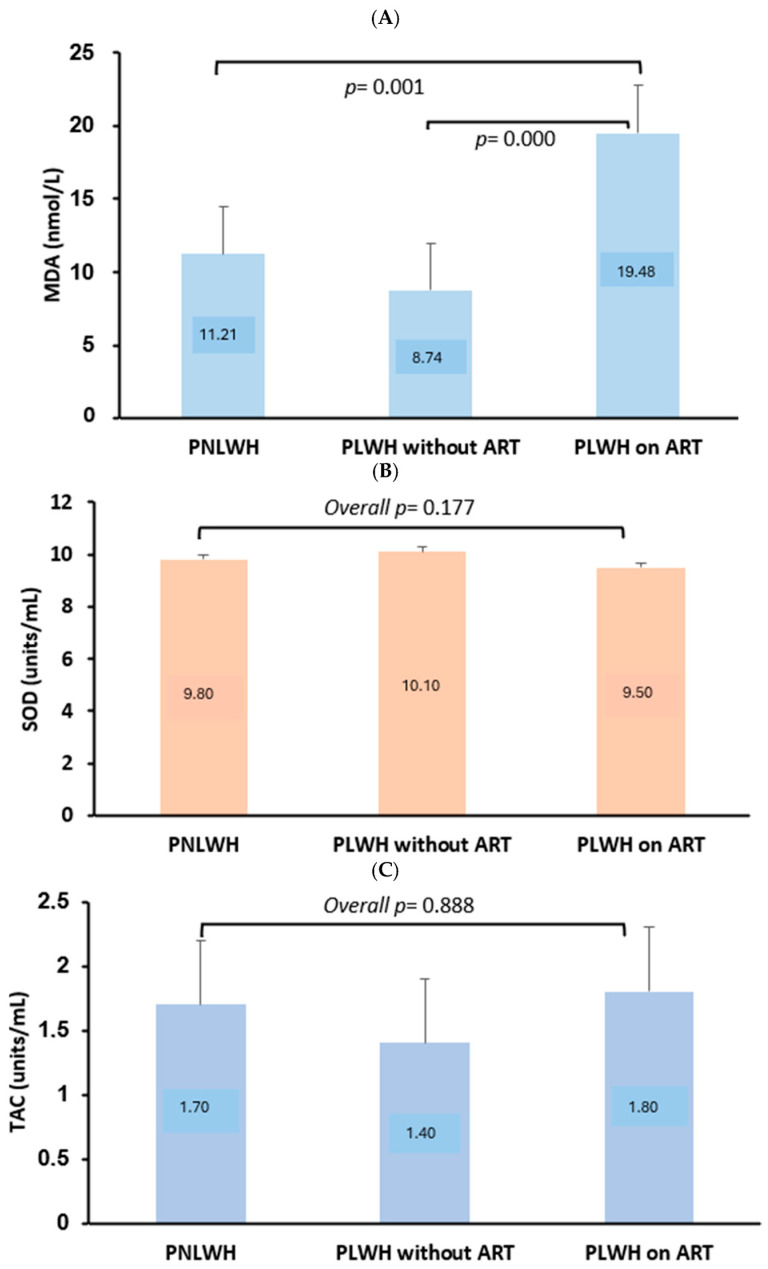
The level of oxidative stress in people living with HIV (PLWH) on antiretroviral therapy (ART), PLWH without ART, and people not living with HIV (PNLWH). (**A**–**C**) depict the levels of malondialdehyde (MDA), superoxide dismutase (SOD) and total antioxidant capacity (TAC), respectively. The MDA levels were significantly different among the groups (*p* < 0.001), with these being higher in PLWH on ART compared to PLWH without ART (*p* = 0.000) and PNLWH (*p* = 0.001) as shown in (**A**). There was no difference in SOD and TAC levels among the groups as shown in (**B**,**C**) (*p* = 0.177 and 0.888, respectively).

Regarding oxidative stress, PLWH on ART had significantly elevated MDA levels [19.48 nmol/L (IQR 11.10–40.48)] compared to PLWH not on ART (*p* = 0.000) and the PNLWH (*p* = 0.001). There were no significant differences in SOD activity or total antioxidant capacity (TAC) among the groups (*p* = 0.177 and *p* = 0.888, respectively) (Figure 1; Table 2).

### 2.2. Comparative Analysis of Oxidative Stress Markers Across Distinct eGFR_cystC_ Stages Within the Study Population

To evaluate the influence of renal function on oxidative stress, we assessed the distribution of MDA and SOD across three cystatin C-based estimated glomerular filtration rate (eGFR_cystC_) categories: ≥90 mL/min/1.73 m^2^ (normal renal function), 60–89 mL/min/1.73 m^2^ (mild dysfunction), and <60 mL/min/1.73 m^2^ (chronic kidney disease).

In the overall study population, MDA levels were significantly different across the eGFR_cystC_ stages (*p* = 0.011), but no significant pairwise differences were noted. In contrast, SOD levels were significantly reduced in individuals with mild renal dysfunction (eGFR_cystC_ 60–89) compared to those with normal function (*p* = 0.020), reinforcing the relevance of antioxidant decline in early renal compromise (Table 2).

In PLWH on ART, MDA levels did not differ significantly across eGFR_cystC_ stages (*p* = 0.227), suggesting no clear association between lipid peroxidation and renal function in this group (Table 2). However, a significant difference in SOD levels was observed (*p* = 0.009). Further analysis revealed that SOD activity was significantly lower in participants with mild renal dysfunction (eGFR_cystC_ 60–89) compared to those with normal kidney function (eGFR_cystC_ ≥ 90, *p* = 0.017), indicating reduced antioxidant defence associated with early renal impairment in this population (Table 2).

Among all PLWH, MDA levels also showed no statistically significant difference across eGFR_cystC_ stages (*p* = 0.076). However, SOD levels were significantly different overall (*p* = 0.038), though post hoc comparisons did not confirm specific pairwise differences. These findings suggest a possible decline in antioxidant status with deteriorating renal function, although not as clearly defined as in the ART subgroup (Table 2).

In Table 3, MDA and SOD levels are compared between two eGFR_cystC_ stages: eGFR_cystC_ ≥ 90 for normal renal function and eGFR_cystC_ < 90 for reduced renal function. The MDA levels showed a significant difference between the two eGFR_cystC_ stages when including all PLWH (U = 1443.00, *n* = 75 and 30, *p* = 0.024), with further comparisons revealing higher levels in eGFR_cystC_ < 90 with a median value of 24.78 nmol/L (IQR 12.38–41.70) compared to eGFR_cystC_ ≥ 90. Further observation in the overall cohort of PLWH showed a significant difference in SOD levels between the two eGFR_cystC_ stages (U = 669.50, *n* = 75 and 30, *p* = 0.012), and the levels were significantly reduced in eGFR_cystC_ < 90, with a median value of 9.20 units/mL (IQR 8.97–9.53) compared to eGFR_cystC_ ≥ 90. In PLWH on ART, MDA levels showed no significant difference between the two eGFR_cystC_ stages (*p* = 0.109); however, SOD levels differed significantly (U = 344.0, *n* = 49 and 25, *p* = 0.022), as shown in Table 3. The SOD levels were significantly lower in eGFR_cystC_ < 90, with a median value of 9.20 units/mL (IQR 8.98–9.48), compared to eGFR_cystC_ ≥ 90 in PLWH on ART.

Table 4 shows a separate comparison of TAC levels between eGFR_cystC_ ≥ 90 and eGFR_cystC_ < 90. This analysis was carried out separately since the subgroup sample sizes for TAC differed from those of MDA and SOD due to limited sample volume. Briefly, there was no significant difference in TAC levels between normal and reduced renal function for the overall study group comprising PLWH (*p* = 1.000), PLWH on ART (*p* = 0.883), PLWH without ART (*p* = 0.883), and PNLWH (*p* = 0.930) (Table 4).

### 2.3. Association Between Oxidative Stress and Renal Function

#### 2.3.1. Spearman Associations Between Oxidative and Renal Function Markers in the Overall PLWH, PLWH on ART and PNLWH

To explore potential associations between oxidative stress and renal function, Spearman rank correlation analyses were performed between oxidative stress markers (MDA, SOD, and TAC) and eGFR_cystC_ across key study subgroups (Table 5).

In PLWH on ART, a significant inverse correlation was observed between MDA and eGFR_cystC_ (r = ‒0.23, 95% CI: ‒0.44 to ‒0.01, *p* = 0.039), indicating that higher lipid peroxidation was associated with modestly lower renal function. Conversely, SOD levels were positively associated with eGFR_cystC_ (r = 0.31, 95% CI: 0.08 to 0.50, *p* = 0.008), suggesting that increases in antioxidant activity may have a modest effect on protecting against renal decline. No significant correlation was observed between TAC and eGFR_cystC_ in this group.

In the overall PLWH population, similar trends were observed. MDA was significantly associated with decreased eGFR_cystC_ (r = ‒0.28, 95% CI: ‒0.42 to ‒0.12, *p* < 0.001), while SOD showed a positive correlation with eGFR_cystC_ (r = 0.20, 95% CI: 0.03 to 0.36, *p* = 0.017). TAC did not demonstrate any significant relationship with renal function in this group.

Among PNLWH, no statistically significant correlations were identified between any of the oxidative stress markers (MDA, SOD, or TAC) and eGFR_cystC_, suggesting a distinct oxidative stress–renal function dynamic in PLWH.

#### 2.3.2. Multiple Linear Regression Analysis for Associations Between Oxidative Stress Markers and eGFR_cystC_ in the Overall PLWH and PLWH on ART Cohorts

##### Multiple Linear Regression Analysis: Oxidative Stress Markers and Renal Function

To determine the independent associations between oxidative stress markers and renal function, multiple linear regression analyses were conducted using eGFR_cystC_ as the dependent variable. This analysis focused on MDA and SOD; TAC was excluded due to limited sample volume. The regression models are adjusted for key covariates, including age, tobacco use, BMI, glucose, and blood pressure (Table 6).

In PLWH on ART, SOD activity showed a statistically significant positive association with eGFR_cystC_. Prior to adjustment, higher SOD levels were associated with improved renal function (β = 2.67, 95% CI: 0.42 to 4.91, *p* = 0.020). This association remained significant after adjustment for age, tobacco use, BMI, glucose, and blood pressure (β = 2.26, 95% CI: 0.09 to 4.44, *p* = 0.042), indicating that enhanced antioxidant defence may independently predict better renal function in this group (Table 6). In contrast, MDA was not significantly associated with eGFR_cystC_ before (*p* = 0.413) or after (*p* = 0.327) adjustment. The model incorporating both MDA and SOD accounted for 10% of the variance in eGFR_cystC_ (R^2^ = 0.104, *p* = 0.020) in this subgroup.

In the overall PLWH population, neither MDA (*p* = 0.361) nor SOD (*p* = 0.085) demonstrated a significant independent association with eGFR_cystC_ after adjustment. However, the combined model still explained a modest, albeit significant, proportion of the variance in renal function (R^2^ = 0.074, *p* = 0.025), suggesting that oxidative stress markers may contribute to renal health in a broader HIV-infected cohort (Table 6).

## 3. Discussion

It remains important to generate data on the relevance of oxidative stress markers in association with renal function in PLWH [17]. Here, MDA, SOD and TAC biomarkers were investigated for their association with renal impairment, revealing complex interactions influenced by ART and renal status. Our findings showed that PLWH on ART exhibit significantly elevated MDA levels compared to those not receiving ART (Figure 1A). These results suggest that long-term ART, particularly regimens including TDF, may contribute to increased oxidative stress. This hypothesis is aligned with previous reports implicating some ART drugs in mitochondrial dysfunction and reactive oxygen species (ROS) generation, which exacerbate oxidative damage [14,18,19,20,21,22]. Despite this, SOD activity and TAC did not differ significantly by ART status, indicating that antioxidant defences may be maintained or compensated despite increased oxidative stress [23,24,25]. However, contrasting findings exist where ART has been reported to reduce antioxidant capacity [26,27]. The duration and consistency of ART may potentially influence the oxidative status in PLWH [28,29].

When examining oxidative stress across renal function stages, increased lipid peroxidation, as reflected by increased MDA levels, was noted in early renal impairment; however, this was found not to escalate further with advancing chronic kidney disease (CKD) stages in PLWH (Table 2). Although clinical studies assessing oxidative markers across eGFR stages in PLWH are generally lacking, substantial preclinical evidence has demonstrated relationships between MDA levels and renal impairment in rat models treated with ART [9,30,31]. Conversely, SOD activity declined significantly in those with mild renal dysfunction, suggesting early depletion of enzymatic antioxidant defences during renal compromise [32,33]. This pattern supports the concept that oxidative stress contributes to the onset of renal dysfunction while antioxidant capacity diminishes concurrently, potentially exacerbating renal injury [31,34,35].

Importantly, multiple linear regression analysis highlighted that among PLWH on ART, higher SOD activity independently predicted better renal function, emphasizing the protective role of antioxidant enzymes in this population [36,37]. In contrast, MDA was not independently associated with renal function decline, suggesting that markers of oxidative damage alone may not directly drive renal deterioration but could act synergistically with other risk factors [38,39].

The lack of significant associations between oxidative stress markers and renal function in PLWH not receiving ART, partly due to the limited sample size, highlights the need for larger studies to clarify these relationships. Additionally, no significant correlations were found in PNLWH, indicating a distinct oxidative stress to renal function interplay in PLWH, likely influenced by HIV infection and ART [18,40,41,42,43].

These results collectively highlight oxidative stress, notably diminished enzymatic antioxidant capacity, as a potential early marker and modifiable factor in renal dysfunction among PLWH. Thus, monitoring oxidative stress markers like SOD, could aid in early detection and prevention of CKD progression in this vulnerable group [5].

Limitations of this study include its cross-sectional design, which precludes causal inferences, and the relatively small sample size and unequal sample sizes across study groups, limiting subgroup analyses. The small sample size of PLWH without ART, which is due to recruitment challenges, further limits the statistical power and generalizability of the findings. More robust recruitment strategies will be employed in future work to achieve adequate statistical power for all groups. The findings may also be limited by the length of time participants have been on ART. Despite these constraints, this study provides valuable insights into the oxidative stress to renal function axis in PLWH, particularly in the context of ART. The findings represent crucial pilot data which warrant further future investigations in adequately powered studies with comprehensive analysis of oxidative stress, including additional markers such as catalase activity, glutathione peroxidase, glutathione reductase, glutathione S-transferase or reduced glutathione concentration. This additional analysis was not feasible within the current study, as the available biological samples and data were limited to the assays originally planned (MDA, SOD, and TAC). The preliminary findings from this pilot data serve as a guide for future research directions.

## 4. Materials and Methods

### 4.1. Study Design, Ethics and Sample Size

This study was conducted at Mankweng Hospital in the Capricorn district, Limpopo Province, South Africa. Approval for the study was obtained from the University of Limpopo Ethics Committee (project number TREC/105/2023: PG), 4 April 2023. This was a comparative cross-sectional study involving adults (≥18 years; *n* = 151), grouped as follows: PLWH on ART (*n* = 80) and those not on treatment (*n* = 27), while people not living with HIV (PNLWH) were also included (*n* = 44). Patients were grouped regardless of age, with the following exclusions: “pre-existing renal disease”, “hypertension”, “diabetes”, and/ or co-infections such as “hepatitis B and C” based on medical record screening. Patients who were taking other medications that are known to affect renal function, such as cimetidine or nonsteroidal anti-inflammatory drugs, were also excluded. The random sampling method was employed to recruit and enroll participants. Briefly, a sample size of 151 subjects was predetermined using the Cochran formula with a 5% margin of error and a 95% confidence level, considering an estimated HIV prevalence of 11% in the Limpopo Province [44,45]. The data collection followed an organized approach, where informed consents were first requested from participants, followed by interviews to collect relevant demographic and medical data. The study design is strengthened or validated by ongoing research that has been previously published [46,47]. The study was conducted over a period of 12 months.

### 4.2. Medical Records, Blood Pressure and Anthropometric Measurements

The medical records were screened to retrieve data that could assist in ascertaining the eligibility of participants, such as HIV status and pre-existing conditions, and other relevant data such as ART status, specific ART regimen, duration on regimen, and lifestyle factors such as tobacco smoking status. Systolic and diastolic blood pressure were measured using an automated blood pressure monitor (OMRON Healthcare, Kyoto, Japan) according to the manufacturer’s protocol. Anthropometric parameters, including weight and height, were measured using an automated weight scale (Pee Pee Electricals, Delhi, India) and a portable Seca Stadiometer (Seca, Hamburg, Germany), adhering to the manufacturer’s instructions. Body mass index (BMI) was calculated as the individual’s weight in kilograms (kg) divided by the square of their height in meters (m).

### 4.3. Blood Collection, HIV Status and CD4+ T-Cell Count Determination

Five milliliters (5 mL) of venous blood were drawn from each participant by a qualified healthcare professional. The blood sample was collected using red-top and ethylenediaminetetraacetic acid-containing blood collection tubes (Beckton Dickinson, Franklin Lakes, NJ, USA), followed by centrifuging at 1500× *g* for 15 min using an Allegra X-30 Series benchtop centrifuge (Beckman Coulter, Brea, CA, USA) to obtain serum and plasma samples. To ascertain the HIV status, an HIV test was conducted using the Alere Determine HIV-1/2 Ag/Ab Combo test kit (Abbott Diagnostics Medical Co., Ltd., Tokyo, Japan), guided by the manufacturer’s protocol. The CD4+ T-cell counts were determined for all PLWH using the Alere PIMA analyser (Alere Technologies GmbH, Jena, Germany). Samples were stored at −80 °C until assayed for oxidative stress, glucose levels and kidney function. The principles of the Declaration of Helsinki [48] were adhered to throughout the data collection process.

### 4.4. Measurements for Markers of Oxidative Stress, Glucose Levels, and Kidney Function

The markers of oxidative stress measured in serum samples included MDA quantified using a colorimetric assay kit (Catalog number: NBP3-24519; Novus Biologicals, Centennial, CO, USA), and SOD activity quantified using the colorimetric assay kit (Catalog number: CS0009; Sigma-Aldrich, St Louis, MO, USA). The average intra-plate coefficient of variation was 4.9% and <9 % for MDA and SOD, respectively. Total antioxidant capacity (TAC) was determined in serum using the TAC kit (Catalog number MAK187; Sigma-Aldrich, St Louis, MO, USA) and the average intra-plate coefficient of variation was less than 3%. The assays were performed following the manufacturer’s protocol. Glucose was analysed in serum using the Cobas Integra^®^ 400 plus autoanalyzer (Roche Diagnostics, Indianapolis, IN, USA) as per the manufacturer’s protocol. For renal function, plasma cystatin C was measured using a bead-based human multiplex immunoassay using Luminex^®^ xMAP^®^ technology (Catalogue number: HKI6MAG-99K; Merck Millipore, Burlington, MA, USA) guided by the manufacturer’s instructions. This was followed by determining eGFR by employing the Chronic Kidney Disease-Epidemiology Collaboration (CKD-EPI) formula [49]. Briefly, the cystatin C-based estimated glomerular filtration rate (eGFR_cystC_) was categorized into stages, including normal renal function (eGFR_cystC_ ≥ 90 mL/min/1.73 m^2^), mild renal dysfunction (eGFR_cystC_ 60–89 mL/min/1.73 m^2^), and moderate-to-severe renal dysfunction and renal failure, also known as chronic kidney disease (CKD) (eGFR_cystC_ < 60 mL/min/1.73 m^2^). The eGFR was further characterized into two categories: eGFR_cystC_ ≥ 90 mL/min/1.73 m^2^ for normal renal function and eGFR_cystC_ < 90 mL/min/1.73 m^2^ indicating reduced renal function. The National Kidney Foundation Kidney Disease Outcomes Quality Initiative (NKF KDOQI) guidelines were used to classify eGFR_cystC_ [50].

### 4.5. Statistical Analysis

The data obtained was analyzed using the IBM Statistical Package for the Social Sciences (SPSS) statistics software (version 29) (International Business Machines Corporation, New York, NY, USA). All scale variables were subjected to the Shapiro–Wilk test of normality to determine whether the data was normally or non-normally distributed. The variables were found to be non-normally distributed and thus, independent samples Mann–Whitney U (also known as the Wilcoxon rank-sum test) and Kruskal–Wallis tests were employed to compare median differences between groups. The chi-square test was used to obtain frequencies and percentages for categorical data. The associations between oxidative stress and renal function markers were assessed using Spearman correlation analysis and multiple linear regression. The *p*-value was set at <0.05 for statistical significance.

## 5. Conclusions

Oxidative damage accompanied by the failure of the body’s primary antioxidant defences may be present at the early onset of renal function decline in PLWH. Superoxide dismutase, as an antioxidant defence enzyme, may be a key contributor to renal health in PLWH on ART. Future longitudinal studies with larger cohorts should clarify causal pathways, evaluate interventions to reduce oxidative stress, and assess the value of ‘oxidative markers’ in predicting renal outcomes in PLWH.

## Figures and Tables

**Table 1:** Baseline data, including demographic and clinical characteristics of the study population.

		PLWH on ART *n* = 80	PLWH Without ART *n* = 27	PNLWH *n* = 44	*p*-Value
**Age (years)**		42.50 (36.25–49.75)	38.00 (28.00–45.00)	32.00 (25.00–45.50)	**<0.001**
**Sex *n* (%)**					
	Female	56.00 (70.00)	16.00 (59.30)	29.00 (65.90)	0.583
	Male	24.00 (30.00)	11.00 (40.70)	15.00 (34.10)	
**Tobacco smoking *n* (%)**					
	Smoking	20.00 (25.00)	6.00 (22.20)	7.00 (15.90)	0.503
	Not smoking	60.00 (75.50)	21.00 (77.80)	37.00 (84.10)	
**Duration on ART (years)**					
	<3 years	28.00 (37.30)	-	-	-
	≥3 years	47.00 (62.70)	-	-	
**Specific regimen *n* (%)**					
**TDF + FTC + EFV**		61.00 (77.20)	-	-	
**ABC + 3TC + EFV**		1.00 (1.30)	-	-	
**AZT + 3TC + NVP**		2.00 (2.50)	-	-	
**AZT + 3TC + LPV/r**		14.00 (17.50)	-	-	
**TDF + FTC + LPV/r**		1.00 (1.30)	-	-	
**CD4+ T-cell count (cells/µL)**		441.00 (247.50–566.00)	228.00 (104.00–389.00)	-	**0.004**
**BMI (kg/m^2^)**		26.12 (22.03–39.87)	23.27 (20.49–26.67)	27.00 (22.28–32.81)	**0.043**
**SBP (mmHg)**		119.00 (107.00–132.00)	119.00 (107.00–127.00)	115.00 (110.00–126.00)	0.791
**DBP (mmHg)**		75.00 (69.00–81.75)	75.00 (69.00–80.00)	74.50 (67.25–79.00)	0.690
**Serum glucose (mmol/L)**		5.20 (4.60–5.80)	4.70 (4.40–5.20)	4.80 (4.40–5.58)	0.069

Pairwise comparison: Age is significantly different between PLWH on ART and PNLWH, with adjusted *p* = 0.000. BMI is significantly different between PLWH without ART and PNLWH, with adjusted *p* = 0.039. Abbreviations: ABC: abacavir; ART: antiretroviral therapy; AZT: zidovudine; BMI: body mass index; CD4+: cluster of differentiation 4 positive; DBP: diastolic blood pressure; EFV: efavirenz; FTC: emtricitabine; LPV/r: ritonavir-boosted lopinavir; NVP: nevirapine; PLWH: people living with HIV; PNLWH: people not living with HIV; SBP: systolic blood pressure; TDF: tenofovir disoproxil fumarate; 3TC: Lamivudine. Notes: results expressed as median (interquartile range), and as frequency and percentage. A bold *p*-value indicates significance.

**Table 2:** Markers of oxidative stress among three eGFR_cystC_ stages in the overall population, overall PLWH and, PLWH on ART.

	Overall Study Population			
	eGFR_cystC_ ≥ 90	eGFR_cystC_ 60–89	eGFR_cystC_ < 60	*p*-Value
**MDA (nmol/L)**	11.59 (6.32–22.88)	22.12 (11.68–33.88)	37.98 (11.93–50.03)	**0.011**
**SOD (units/mL)**	9.86 (9.20–11.67)	9.20 (8.96–9.48)	9.26 (9.0–9.85)	**0.009**
	**Overall PLWH**			
**MDA (nmol/L)**	12.12 (6.83–28.69)	24.56 (12.67–37.69)	39.05 (11.50–51.15)	0.076
**SOD (units/mL)**	9.86 (9.11–11.24)	9.19 (8.92–9.46)	9.28 (8.95–9.93)	**0.038**
	**PLWH on ART**			
**MDA (nmol/L)**	16.17 (8.80–37.72)	25.00 (14.89–38.34)	41.49 (12.78–51.33)	0.227
**SOD (units/mL)**	9.74 (9.21–11.69)	9.19 (8.98–9.41)	9.26 (8.91–9.69)	**0.009**

SOD significant between eGFR_cystC_ ≥ 90 and eGFR_cystC_ 60–89 for overall population (*p* = 0.020). SOD significant between eGFR_cystC_ ≥ 90 and eGFR_cystC_ 60–89 for PLWH on ART (*p* = 0.017). Results are expressed as median (interquartile range). Abbreviations: ART: antiretroviral therapy; eGFR_cystC_: cystatin C-based estimated glomerular filtration rate; MDA: malondialdehyde; PLWH: people living with HIV; SOD: superoxide dismutase. *p*-value indicates the significance level. A bold *p*-value indicates significance at *p* < 0.05.

**Table 3:** Markers of oxidative stress, including malondialdehyde and superoxide dismutase, between eGFR_cystC_ ≥ 90 (normal renal function) and eGFR_cystC_ < 90 (reduced renal function).

	Overall Study Population		
	eGFR_cystC_ ≥ 90	eGFR_cystC_ < 90	*p*-Value
MDA (nmol/L)	11.59 (6.32–22.88)	23.52 (11.93–39.52)	**0.003**
SOD (units/mL)	9.86 (9.21–11.67)	9.21 (8.97–9.53)	**0.002**
	**Overall PLWH**		
MDA (nmol/L)	12.12 (6.83–28.69)	24.78 (12.38–41.70)	**0.024**
SOD (units/mL)	9.86 (9.11–11.24)	9.20 (8.97–9.53)	**0.012**
	**PLWH on ART**		
MDA (nmol/L)	16.17 (8.80–37.72)	27.17 (13.97–43.93)	0.109
SOD (units/mL)	9.74 (9.21–11.69)	9.20 (8.98–9.48)	**0.002**
	**PLWH without ART**		
MDA (nmol/L)	8.64 (4.85–12.44)	10.42 (3.78–10.42)	0.933
SOD (units/mL)	10.01 (8.72–10.80)	10.16 (8.16)	0.969
	**PNLWH**		
MDA (nmol/L)	10.72 (4.49–19.62)	16.91 (6.52–33.12)	0.351
SOD (units/mL)	9.87 (9.32–11.89)	9.24 (9.03–12.94)	0.417

Results are expressed as median (interquartile range). Abbreviations: ART: antiretroviral therapy; eGFR_cystC_: cystatin C-based estimated glomerular filtration rate; MDA: malondialdehyde; PLWH: people living with HIV; PNLWH: people not living with HIV; SOD: superoxide dismutase. *p*-value indicates the significance level. Bold *p*-value indicates significance at *p* < 0.05.

**Table 4:** Total antioxidant capacity between eGFR_cystC_ ≥ 90 (normal renal function) and eGFR_cystC_ < 90 (reduced renal function).

	Overall Study Population		
	eGFR_cystC_ ≥ 90	eGFR_cystC_ < 90	*p*-Value
TAC (nmol/µL)	1.69 (1.40–2.24)	1.67 (1.22–2.36)	0.914
	**Overall PLWH**		
TAC (nmol/µL)	1.65 (1.38–2.27)	1.67 (1.27–2.29)	1.000
	**PLWH on ART**		
TAC (nmol/µL)	1.76 (1.35–2.09)	1.77 (1.24–2.31)	0.883
	**PLWH without ART**		
TAC (nmol/µL)	1.45 (1.33–5.55)	1.42 (1.42–1.42)	0.883
	**PNLWH**		
**TAC (nmol/µL)**	1.72 (1.49–2.22)	1.85 (1.17–3.47)	0.930

Results are expressed as median (interquartile range). Abbreviations: ART: antiretroviral therapy; eGFR_cystC_: cystatin C-based estimated glomerular filtration rate; PLWH: people living with HIV; PNLWH: people not living with HIV. *p*-value indicates the significance level.

**Table 5:** Spearman rank associations between oxidative stress markers, including malondialdehyde (MDA), superoxide dismutase (SOD) and total antioxidant capacity (TAC), and eGFR_cystC_.

Overall PLWH			
	Correlation Coefficient (r)	*p*-Value	95% Confidence Interval (Lower Bound; Upper Bound)
MDA-eGFR_cystC_	−0.28 **	**<0.001**	−0.42; −0.12
SOD-eGFR_cystC_	0.20 *	**0.017**	0.03; 0.36
TAC- eGFR_cystC_	0.09	0.422	−0.14; 0.32
**PLWH on ART**			
MDA-eGFR_cystC_	−0.23 *	**0.039**	−0.44; −0.01
SOD-eGFR_cystC_	0.31 **	**0.008**	0.08; 0.50
TAC- eGFR_cystC_	−0.09	0.628	−0.42; 0.27
**PLWH without ART**			
MDA-eGFR_cystC_	−0.16	0.443	−0.53; 0.26
SOD-eGFR_cystC_	0.10	0.629	−0.32; 0.49
TAC- eGFR_cystC_	0.57	0.067	−0.07; 0.88
**PNLWH**			
MDA-eGFR_cystC_	−0.20	0.191	−0.48; 0.11
SOD-eGFR_cystC_	0.07	0.659	−0.24; 0.37
TAC- eGFR_cystC_	0.14	0.464	−0.24; 0.48

Abbreviations: ART: antiretroviral therapy; eGFR_cystC_: cystatin C-based estimated glomerular filtration rate; MDA: malondialdehyde; PLWH: people living with HIV; PNLWH: people not living with HIV; SOD: superoxide dismutase; TAC: total antioxidant capacity. *p*-value indicates significance level. ** Correlation is significant at the 0.01 level. * Correlation is significant at the 0.05 level.

**Table 6:** Multiple linear regression analysis of the associations between oxidative stress markers and eGFR_cystC_ as a dependent variable in the overall PLWH and PLWH on ART cohorts.

Subgroup	Unadjusted (R^2^ = 0.074, *p* = 0.025)			
**Overall PLWH**		**β**	***p*** **-value**	**95% confidence interval** **(Lower bound; upper bound)**
	MDA	−0.27	0.138	−0.63; 0.09
	SOD	2.13	0.061	−0.10; 4.36
	**Adjusted**			
	MDA	−0.16	0.361	−0.51; 0.19
	SOD	1.87	0.085	−0.26; 4.00
	**Unadjusted (R^2^ = 0.104, *p* = 0.020)**			
**PLWH on ART**		**β**	***p*** **-value**	**95% confidence interval** **(Lower bound; upper bound)**
	MDA	−0.16	0.413	−0.55; 0.23
	SOD	2.67	**0.020**	0.42; 4.91
	**Adjusted**			
	MDA	−0.19	0.327	−0.57; 0.10
	SOD	2.26	**0.042**	0.09; 4.44

Variables adjusted in the model include age, tobacco use, BMI, glucose, and blood pressure. Abbreviations: β: correlation coefficient; ART: antiretroviral therapy; eGFR_cystC_: cystatin C-based estimated glomerular filtration rate; MDA: malondialdehyde; PLWH: people living with HIV; SOD: superoxide dismutase. *p*-value indicates the level of significance. A bold *p*-value indicates significance at *p* < 0.05.

## Data Availability

The original contributions presented in this study are included in the article. Further inquiries can be directed to the corresponding author.

## Abbreviations

ART: antiretroviral therapy; BMI: body mass index; CD4+: cluster of differentiation 4 positive; CKD: chronic kidney disease; CKD-EPI: chronic kidney disease epidemiology collaboration; eGFR_cystC_: estimated glomerular filtration rate; HIV: human immunodeficiency virus; MDA: malondialdehyde; PLWH: people living with HIV; PNLWH: people not living with HIV; SPSS: statistical package for the social science; SOD: superoxide dismutase; 3TC: lamivudine, ABC: abacavir; AZT: zidovudine; EFV: efavirenz; FTC: emtricitabine; LPV/r: ritonavir-boosted lopinavir; NVP: nevirapine; TAC: total antioxidant capacity; TDF: tenofovir disoproxil fumarate.
